# Effect of digital chemotherapy extravasation education based on Orem’s self‐care theory in cancer patients: A quasi-experimental study

**DOI:** 10.1371/journal.pone.0343000

**Published:** 2026-02-23

**Authors:** Hang Thi Nguyet Ho, Trang Thi Kieu Duong, Nguyet Thi Tran, Phuong Thi Anh Nguyen, Trang Thi Thuy Ho, Duc Nu Minh Ton

**Affiliations:** 1 Hue University of Medicine and Pharmacy, Hue University, Hue, Vietnam; 2 Hue University of Medicine and Pharmacy Hospital, Hue, Vietnam; 3 Da Nang University of Medical Technology and Pharmacy, Danang, Vietnam; Longgang Otorhinolaryngology Hospital & Shenzhen Key Laboratory of Otorhinolaryngology, Shenzhen Institute of Otorhinolaryngology, CHINA

## Abstract

**Purpose:**

Digital health education is crucial in preventing and managing chemotherapy extravasation among cancer patients. We aimed to evaluate the effectiveness of a digital chemotherapy extravasation educational intervention on knowledge, self-care abilities, and self-efficacy in cancer patients.

**Methods:**

A quasi-experimental longitudinal design was conducted with 80 cancer patients at Hue University of Medicine and Pharmacy Hospital, Vietnam, from September 2022 to November 2023. The control group (n = 40) received routine health education on chemotherapy extravasation, while the experimental group (n = 40) participated in an extravasation education program based on Orem’s Self-Care Theory and utilized digital technology. This study assessed knowledge levels, self-care abilities, self-efficacy in preventing and managing chemotherapy extravasation, and the effectiveness of digital technology applications at three different time points. Chi-square tests, independent t-tests, and Analysis of Variance with Repeated Measures were employed to analyze the data.

**Results:**

There were statistically significant differences in knowledge (F = 16.24, *p* < 0.001), self-care abilities (F = 35.37, *p* < 0.001), and self-efficacy (F = 46.27, *p* < 0.001) between the intervention and control groups over time, with the intervention group showing greater improvements across all outcomes. Qualitative findings revealed three key categories: moving forward to self-care readiness, promoting emotional resilience, and enhancing a positive care environment. Patients perceived the digital technology applications as highly effective, especially in terms of perceived usefulness and ease of use, and showed significant changes across time (F = 9.93, *p* < 0.001).

**Conclusion:**

Digital chemotherapy extravasation education based on Orem’s Self-Care Theory offers an effective approach that enhances cancer patients’ knowledge while strengthening their self-care. Therefore, healthcare providers should integrate digital education into routine patient care to enhance self-efficacy, reduce complications, and support better treatment outcomes.

## Introduction

Cancer poses a significant health concern in humans globally. According to GLOBOCAN in 2022, it is estimated that nearly 19,980,000 new cases of cancer and over 9,700,000 cancer-related deaths were found worldwide [1]. According to this report, there have been over 180,000 new cases of cancer and over 120,000 cancer-related deaths in Vietnam. Chemotherapy is the most common and fundamental treatment method for cancer, with over one million intravenous lines placed to administer chemotherapy daily worldwide [2,3]. Extravasation is a potential complication of cancer chemotherapy, with estimated rates ranging from 0.1% to 6% in various studies [4,5]. It refers to the leakage of a medication or infusion solution from a vein or infusion line into the adjacent tissues, where it can cause local irritation or injury [4,6]. The extent of damage depends on the toxicity of the compound and the amount of drug released, leading to skin irritation such as swelling, pain, and possible necrosis [4,6]. Therefore, reducing effect of chemotherapy extravasation is crucial to minimize patient damage and complications.

Prevention and early detection of chemotherapy extravasation are essential strategies to minimize complications and improve patient outcomes [4,7]. Patient’s ability to engage in self-care plays a crucial role in recognizing early signs and symptoms, ensuring timely intervention, and reducing the risk of complications [8,9]. Nurse-led interventions are recognized as effective strategies to support patients during hospitalization and facilitate their transition to home [8,10,11]. These interventions not only encompass patient care and complication prevention but also focus on empowering patients through education, skill-building, and continuous support [4,6]. By integrating these comprehensive strategies, patients become more confident in managing their condition, leading to improved self-care and overall treatment outcomes.

Orem’s Self-care theory, developed by Dorothea Orem, proposes that nursing care aims to help individuals maintain or restore their ability to perform self-care activities that support health, recovery, and well-being [12,13]. The theory emphasizes that when individuals are unable to meet their own self-care needs due to illness or limitations, nurses assume a supportive or educational role to help them regain independence [12,13]. Orem’s Self-care theory is widely recognized and applied in nursing practice, especially in developing patient health education programs to enhance self-care and treatment adherence [12,13]. Previous studies highlighted that self-care education programs enhanced understanding of the disease and its management, leading to better patient compliance, reduced complications, and improved quality of life among cancer patients [9,14,15]. Moreover, most health education programs rely on face-to-face interactions for patient learning and engagement. However, with the rapid advancement of digital technology, healthcare is undergoing a significant transformation. Digital platforms are increasingly being integrated into patient education programs. These innovations enhance accessibility, provide continuous support, and empower patients with real-time information, ultimately improving self-care and patient outcomes [16,17].

Therefore, this study aimed to investigate the effect of digital chemotherapy extravasation education based on Orem’s self-care model on the knowledge, self-care abilities, and self-efficacy of patients undergoing chemotherapy. The findings of the study can help increase the quality of care among cancer patients undergoing chemotherapy.

## Materials and methods

### Study design and participants

**Fig 1:**
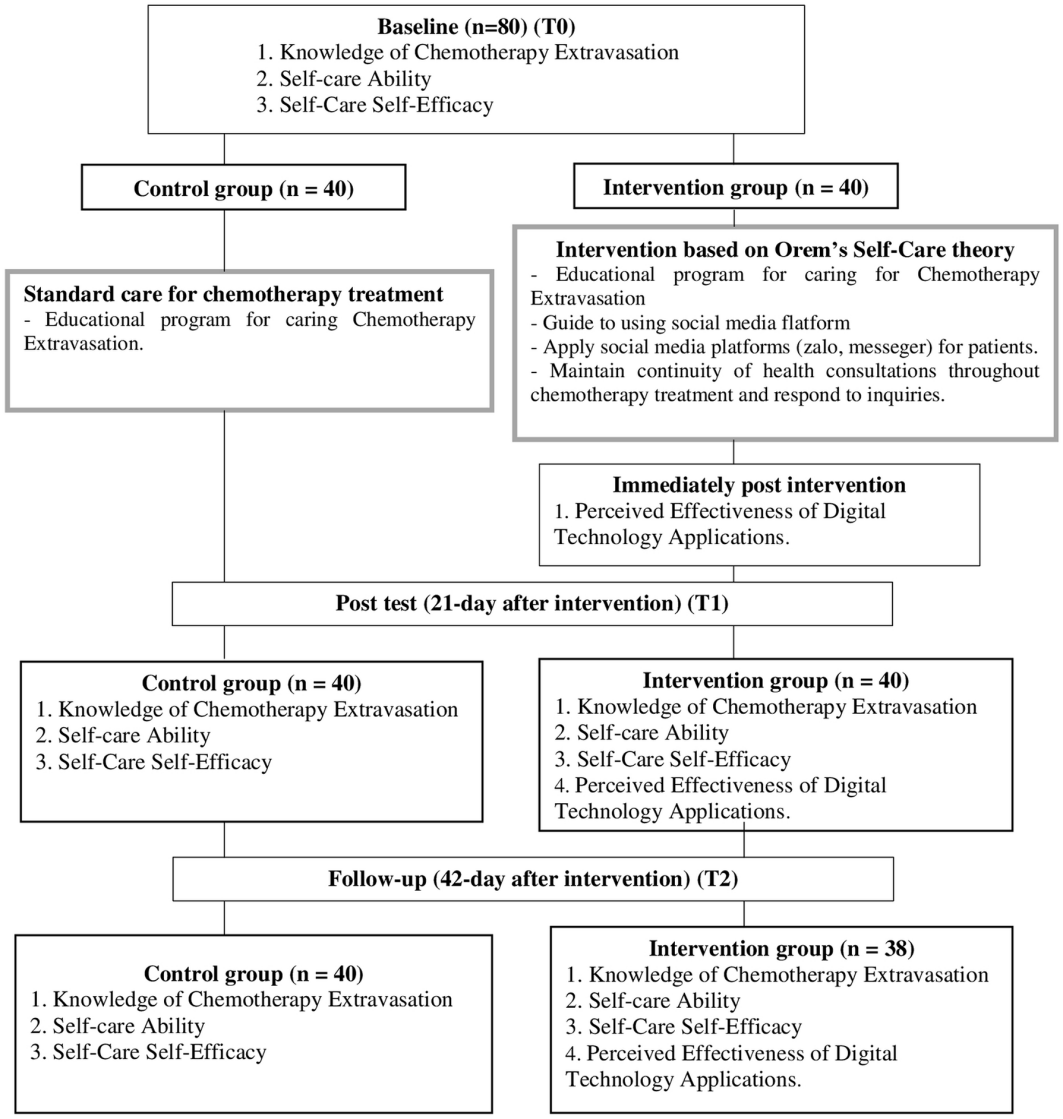
Participants flow through the trial.

This study employed a concurrent triangulation mixed-methods design, integrating a quasi-experimental longitudinal approach with in-depth interviews to enhance validity through methodological triangulation. Data were collected from September 1st, 2022, to November 20^th^, 2023, allowing for both quantitative assessment over time and qualitative exploration of participant experiences. The data were collected at baseline before the intervention (T0), after the 21-day intervention (T1), and after the 42-day intervention follow-up (T2) to evaluate the effects of the intervention (Fig 1).

This study was conducted at Hue University of Medicine and Pharmacy Hospital in central Vietnam. A total of 80 participants were screened for enrolment in the study. The sample size was calculated using G*Power version 3.1.9.4 software, effect size = 0.44, α = 0.25, and power = 0.90, and 80 participants were required for the calculation. Cancer patients were recruited and allocated to the experimental (n = 40) or control group (n = 40). Of these, 40 participants in the experimental group completed the program, and 2 were lost to follow-up because they changed to subcutaneous port catheters. The non-random sampling method was used for this study, in which we selected the control group first to collect the data at three time points as mentioned above, and then the experimental group was recruited. This approach was chosen for ethical and practical reasons: random allocation was not feasible because the intervention involved an educational program that could not be withheld from eligible participants once it became available. In addition, the sequential recruitment helped prevent information contamination between participants in the control and intervention groups who were treated in the same hospital setting. This design ensured adequate participant numbers in each group while maintaining the integrity of the intervention delivery.

The inclusion criteria for this study were patients with cancer who were between 18 and 80 years old, received chemotherapy, had medical records in the oncology department, were capable of understanding and communicating in Vietnamese, and were willing to participate in the study. Patients who experienced extravasation and were transferred from another department were excluded, as well as critically ill patients with limited ability to participate in research due to mental or physical health. Additionally, patients who developed extravasation or ulceration related to a central venous catheter or subcutaneous port catheter were excluded because these devices involve different mechanisms, management protocols, and risk factors compared with peripheral intravenous infusions. This study, therefore, focused exclusively on extravasation events associated with peripheral intravenous chemotherapy administration, where nursing techniques, patient education, and self-care behaviors play a more direct role in prevention and management.

Moreover, in-depth interviews were conducted with six participants who attended digital chemotherapy extravasation program. Additionally, a focus group was held with three nurses who participated in this program.

### Quantitative measures

The study’s outcomes were assessed at baseline, 21-day intervention, and 42-day follow-up after receiving the study intervention. Instruments were used, including:

*Knowledge of chemotherapy extravasation:* The instrument, which assessed knowledge of the prevention and management of chemotherapy extravasation, was developed by researchers. The tool included 28 items with subscales: Fundamentals of Chemotherapy Extravasation (8 items), Symptoms and Prevention of Chemotherapy Extravasation (8 items), and Management of Chemotherapy Extravasation (12 items). The scale was developed mainly based on guidelines of the prevention and management of chemotherapy extravasation [4,18]. All knowledge variables were closed-ended questions. A score of one was given for each correct answer and zero for each incorrect or missed answer. The total possible score ranged from 0 to 28, with a higher score indicating a better knowledge of the prevention and management of chemotherapy extravasation.

*Self-care ability:* Self-care ability tool was modified based on the Self-care of chronic illness inventory of Riegel et al [19] to assess the self-care ability of cancer patients. It includes recognizing chemotherapy extravasation symptoms (1 item), prevention and management of chemotherapy extravasation (8 items), and implementation of management of chemotherapy extravasation (1 item). The instrument consists of 10 items with a 5-point Likert scale, 1 = “completely unable to perform” to 5 = “highly able to perform”. The total possible score ranges from 10 to 50, with the higher the score, the higher the levels of self-care ability.

*Self-care self-efficacy:* The Self-Care Self-Efficacy tool was modified by the authors based on a subscale of the Self-care of chronic illness inventory of Riegel et al [19], which refers to the patient’s perspective on their ability to effectively manage chemotherapy extravasation to minimize adverse outcomes. The instrument consists of 10 items with a 5-point Likert scale, where 5 means “Extremely Confident” and 1 means “Not Confident”. The total possible score ranges from 10 to 50, with higher scores indicating higher levels of self-efficacy in self-care and subjective presence of ability.

*Perceived effectiveness of digital technology applications:* was developed by the authors based on the mHealth app usability questionnaire [20]. The tool is self-administered and consists of four separate scales: perceived usefulness (3 items), perceived ease of use (3 items), attitude toward using (2 items), and trust (2 items). Each item is scored on a 5-point Likert scale, where 1 = “Strongly disagree” and 5 = “Strongly agree”, then the lowest score equals 10 and the highest score equals 50. Higher scores indicate a greater perceived effectiveness of digital technology applications in providing health information.

Prior to the main data collection, the instruments were evaluated for content validity and reliability. They were first reviewed by three experts from the Faculty of Nursing and Medicine to assess their relevance, clarity, comprehensiveness, and applicability. The item-level Content Validity Index (I-CVI) values ranged from 0.75 to 1.00, while the scale-level Content Validity Index (S-CVI) was 0.95 for the knowledge of chemotherapy extravasation scale. The corresponding S-CVI values for the self-care ability scale, the self-care self-efficacy scale, and the perceived effectiveness of digital-technology application tools were 0.73 (I-CVI range: 0.50–0.75), 0.75 (I-CVI range: 0.50–1.00), and 0.75 (I-CVI range: 0.50–1.00), respectively.

The pilot study was subsequently conducted to further assess the clarity and feasibility of the tools before their use in the main study. This tool obtained satisfactory psychometric properties and reliability (internal consistency of Cronbach’s alpha 0.94, 0.72, 0.75, and 0.82, respectively), indicating high internal consistency.

### Qualitative measures

In-depth interviews were conducted to thoroughly explore the effects of the program. Semi-structured questions were used to guide the discussions, including: “Can you describe your experience attending the program?”, “How has the program supported you?”, and “Do you have any additional comments about your experience?” These questions focused on participants’ self-perception of health, knowledge of chemotherapy extravasation, attitudes toward self-care, and the overall impact of the program on their well-being.

### The development of digital chemotherapy extravasation education program

#### Control group.

Participants assigned to the control group were provided with the standard care offered by clinics throughout their chemotherapy treatment. The routine nursing procedures included admission nursing, basic nursing, psychological nursing, medication guidance, diet and health education, and discharge instructions. This care encompassed the distribution of an educational pamphlet and the provision of verbal instructions by healthcare professionals during their clinic appointments.

#### Experimental Group.

The proposed intervention is designed to harness Orem’s Self-Care Theory to empower chemotherapy patients with the necessary knowledge and skills to prevent, recognize, and manage chemotherapy extravasation. This comprehensive educational program comprises modules covering the fundamental aspects of chemotherapy extravasation, prevention strategies, symptom recognition, and immediate and long-term management. The program is structured into several modules, commencing with the facilitation of patient connectivity with healthcare providers through social media platforms such as Zalo (Table 1). The content has undergone meticulous review and revision by experts in the field to ensure its accuracy. Furthermore, health material has been transformed into visually engaging posters and informative videos through artificial intelligence technology.

**Table 1:** Content of digital chemotherapy extravasation educational training.

	Content
Session 1	Understanding Chemotherapy Extravasation Definition and causes Risk factors
Session 2	Prevention Strategies Importance of proper intravenous (IV) technique Tips for patients to monitor their IV sites
Session 3	Recognizing Symptoms Early signs and symptoms of extravasation Visual aids and symptom checklists
Session 4	Immediate Management Steps to take if extravasation is suspected When to seek professional help
Session 5	Long-term Management and Care Physical therapy and rehabilitation if needed
Session 6	Emotional and Psychological Support Guide to Using Social Media Platforms for Health Communication

The program underscores the significance of peer support by directing patients to identify and join pertinent support groups and participate in group discussions and activities. Information sharing is another pivotal element, featuring guidelines for the secure transmission and reception of educational materials and personal health updates. The program incorporates QR codes to sustain health consultations during chemotherapy treatment. Additionally, the program provides guidance to patients on the effective utilization of social media platforms like Zalo for health communication, enabling them to connect with healthcare providers. By integrating QR codes, the program facilitates convenient access to health resources, interactive consultations, and real-time symptom reporting. Overall, the digital component was designed to enhance patients’ self-care competence by enabling early recognition of extravasation signs, promoting timely communication with nurses, and supporting immediate intervention to prevent tissue injury and related complications.

This intervention aims to enhance patients’ self-care capabilities, improve health outcomes, and deliver continuous support during chemotherapy treatment through modern digital tools and a robust educational framework. Moreover, participants were either grouped together or provided with personalized programs based on their individual needs and preferences.

### Data collection

Participants for this study were selected and completed a pretest at the beginning of the program. A posttest was administered 21 days after the intervention, followed by a 42-day follow-up assessment. Additionally, face-to-face semi-structured interviews were held within the 42-day follow-up period. Prior to providing consent and sharing personal information, all participants were informed about the study’s purpose, content, confidentiality agreements, and their right to withdraw at any time. A small incentive, in accordance with survey regulations, was provided to each participant, consisting of a small snack and a drink.

### Data analysis

This study used SPSS 26.0 software (SPSS Inc., Chicago, IL, USA). Descriptive statistics were used to describe the characteristics of the participants. Independent samples t-tests were applied to compare continuous variables between groups. Categorical variables were analyzed using Pearson’s chi-square test. Repeated measures ANOVA was used to assess changes over time between groups. A p-value < 0.05 was considered statistically significant.

To analyze the qualitative data, Microsoft Excel was utilized for content analysis. All interviews were transcribed manually. Two of the authors analyzed these interviews qualitatively using the content analysis method through four stages: decontextualization, recontextualization, categorization, and compilation [21]. The researchers meticulously reviewed the transcribed text multiple times to identify meaning units. These meaning units were further condensed, and relevant codes were extracted. Subsequently, the codes were organized into subcategories, and the items were summarized to ultimately establish the primary category. The two researchers independently identified the category, and consensus on the organization of the main category was reached through discussion among the research team members.

### Ethical considerations

This research followed the Declaration of Helsinki, and the research proposal has received approval from the Scientific Research Ethics Committee of Hue University of Medicine and Pharmacy (Reference No. H2022/110, date 07/06/2022). Before participating, all individuals were given detailed information about the study’s objectives, procedures, potential risks, and confidentiality measures. Participants provided written informed consent, confirming their voluntary involvement and their understanding that they could withdraw at any time without consequences. Additionally, this study adhered to ethical guidelines for human research, ensuring data protection and participant anonymity. No animal studies were conducted, and the manuscript does not include any potentially identifiable images or sensitive personal information.

## Results

### Quantitative results

#### Homogeneity Test of General Characteristics and Variables at Baseline.

A total of 80 cancer patients were involved in this study. The majority of participants were female, and they were in stages III and IV of the disease. Participants were diagnosed with various types of cancer, including breast (35%), colorectal (15%), lung (10%), ovarian (7.5%), stomach (7.5%), and other cancers (25%) in the control group, and a similar distribution was observed in the experimental group (Table 2). There was no statistically significant difference in the type of cancer or other general characteristics between the experimental and control groups at baseline (all *p* > 0.05) (Table 2).

**Table 2:** Comparison of sociodemographic and disease variables between two groups before the intervention (N = 80).

Variables		Cont. (n = 40) n (%)	Exp. (n = 40) n (%)	Test	*p*
Age (years)		56,05 ± 9,34	54,60 ± 12,88	(t) 0.58	0.57^a^
Sex	*Male* *Female*	16 (40) 24 (60)	15 (37.5) 25 (62.5)	–	0.82^b^
Educational level	*Illiteracy* *Primary* *Middle school* *High school* *Junior college* *University or higher*	2 (5) 14 (35) 8 (20) 8 (20) 3 (7.5) 5 (12.5)	0 (0) 4 (10) 10 (25) 16 (40) 4 (10) 6 (15)	–	0.051^b^
Living area	*City* *Countryside* *Mountain Region*	18 (45) 20 (50) 2 (5)	22 (55) 17 (42.5) 1 (2.5)	–	0.61^b^
Economic	*<3 million VNĐ* *3-6 million VND* *>6 million VNĐ*	16 (40) 18 (45) 6 (15)	23 (57.5) 12 (30) 5 (12.5)	–	0.28^b^
Diagnosis	*Ovarian Cancer* *Stomach cancer* *Colorectal cancer* *Lung cancer* *Breast cancer* *Other*	3 (7.5) 3 (7.5) 6 (15) 4 (10) 14 (35) 10 (25)	6 (15) 6 (15) 4 (10) 3 (7.5) 15 (37.5) 6 (15)	–	0.61^b^
Stage of disease	*I* *II* *III* *IV*	1 (2.5) 7 (17.5) 18 (45) 14 (35)	1 (2.5) 15 (37.5) 14 (35) 10 (25)	–	0.25^b^
Previous treatment methods	*No previous treatment* *Surgery*	22 (55) 18 (45)	25 (62.5) 15 (37.5)		0.5^b^

^a^ = Independent Sample-t, ^b^ = Pearson-χ^2^.

As shown in Table 3, no significant difference was found in the baseline (before intervention) knowledge of chemotherapy extravasation, self-care ability, and self-care self-efficacy between the two groups (p > 0.05).

**Table 3:** Comparison of knowledge, self-care, and self-efficacy variables between two groups before the intervention (N = 80).

Variables	Cnt. (n = 40)	Exp. (n = 40)	Test	*p*
	Mean ± SD			
**Knowledge of Chemotherapy Extravasation** Fundamentals of Chemotherapy Extravasation Symptoms and Prevention of Chemotherapy Extravasation Management of Chemotherapy Extravasation	**10.73 ± 7.67** 2.08 ± 2.13 2.65 ± 2.14 6.00 ± 4.22	**11.03 ± 7.23** 2.10 ± 2.05 2.88 ± 2.40 6.05 ± 3.76	**−0.18** −0.05 −0.44 −0.06	**0.86**^**c**^ 0.96^c^ 0.66^c^ 0.96^c^
**Self-care Ability** Recognize chemotherapy extravasation symptoms Prevention and management of chemotherapy extravasation Implementation of management of chemotherapy extravasation	**24.38 ± 9.74** 2.13 ± 1.27 20.20 ± 8.65 2.05 ± 1.15	**22.00 ± 10.76** 1.75 ± 0.98 18.48 ± 9.66 1.78 ± 1.03	**1.04** 1.48 0.84 1.13	**0.30**^**c**^ 0.14^c^ 0.40^c^ 0.26^c^
**Self-Care Self-Efficacy**	**2.68 ± 1.16**	**2.25 ± 1.15**	**1.64**	**0.10** ^**c**^

^c^ = t-test.

#### Effect of the digital chemotherapy extravasation educational training.

The analysis using repeated ANOVA with the Greenhouse-Geisser correction revealed statistically significant interactions between group and time for knowledge of chemotherapy extravasation (F = 16.24, p < 0.001) (Table 4). Specifically, on the subscale of fundamental of chemotherapy extravasation, the experimental group displayed a significant improvement from 2.10 ± 2.05 at baseline to 5.68 ± 1.22 at 42-day follow-up, while the control group exhibited a smaller increase from 2.08 ± 2.13 at baseline to 3.13 ± 2.37 at 42-day follow-up. For the symptom and prevention of chemotherapy extravasation, the experimental group increased from 2.88 ± 2.40 at baseline to 5.70 ± 1.51 at 42-day follow-up, whereas the control group increased from 2.65 ± 2.14 at baseline to 5.33 ± 1.59 at 42-day follow-up. On the subscale of symptoms and prevention of chemotherapy extravasation, the F-values for Group (31.13), Time (22.04), and Time x Group (27.93) were all found to be significant (p < 0.001). Regarding the management of chemotherapy extravasation, the experimental group improved from 6.05 ± 3.76 at baseline to 9.50 ± 1.20 at 42-day follow-up, while the control group showed a decrease from 6.00 ± 4.22 at baseline to 5.60 ± 2.99 at 42-day follow-up. The F-values for Group (25.47), Time (13.75), and Time x Group (9.93) were all significant (p < 0.001).

**Table 4:** The comparison of knowledge of prevention and management of chemotherapy extravasation among two groups (N = 80).

Variable (n=80)	Group	Baseline	21 days	42 days	Source	F	*p*
		Mean ± SD					
Knowledge of Prevention and Management of Chemotherapy Extravasation	Cont.	10.73±7.67	12.65±6.11	12.05±6.47	Group Time Time x Group	38.40 31.67 16.24	< 0.001 < 0.001 < 0.001
	Exp.	11.03±7.23	20.55±3.06	20.88±3.02			
Fundamentals of Chemotherapy Extravasation	Cont.	2.08±2.13	2.53±2.11	3.13±2.37	Group Time Time x Group	41.30 61.60 18.36	< 0.001 < 0.001 < 0.001
	Exp.	2.10±2.05	5.58±1.21	5.68±1.22			
Symptoms and Prevention of Chemotherapy Extravasation	Cont.	2.65±2.14	3.18±1.88	5.33±1.59	Group Time Time x Group	31.13 22.04 27.93	< 0.001 < 0.001 < 0.001
	Exp.	2.88±2.40	3.33±1.98	5.70±1.51			
Management of Chemotherapy Extravasation	Cont.	6.00±4.22	6.95±3.34	5.60±2.99	Group Time Time x Group	25.47 13.75 9.93	< 0.001 < 0.001 < 0.001
	Exp.	6.05±3.76	9.95±1.00	9.50±1.20			

Table 5 indicates that the results showed statistically significant interactions between group and time for self-care ability (F = 35.37, p < 0.001), and self-care self-efficacy (F = 46.27, p < 0.001). The experimental group experienced a significant increase in self-care ability, rising from 22.00 ± 10.76 at baseline to 44.10 ± 4.49 at 42 days follow-up. On the other hand, the control group showed a more modest increase from 24.38 ± 9.74 at baseline to 27.28 ± 8.04 at 42 days follow-up. In terms of self-care self-efficacy, the experimental group demonstrated notable improvement, with scores increasing from 2.25 ± 1.15 at baseline to 4.50 ± 0.59 at 42 days follow-up. Meanwhile, the control group’s scores remained relatively stable, changing from 2.68 ± 1.16 at baseline to 2.66 ± 0.80 at 42 days follow-up.

**Table 5:** The comparison of self-care ability and self-efficacy among two groups (N = 80).

Variable	Group	Pretest	21 days	42 days	Source	F	*p*
		Mean ± SD					
**Self-care Ability**	Cont.	24.38 ± 9.74	26.85 ± 9.08	27.28 ± 8.04	Group Time Time x Group	46.93 60.79 35.37	< 0.001 < 0.001 < 0.001
	Exp.	22.00 ± 10.76	39.15 ± 7.32	44.10 ± 4.49			
*Recognize chemotherapy extravasation symptoms*	Cont.	2.13 ± 1.27	2.42 ± 1.11	2.38 ± 0.98	Group Time Time x Group	25.85 48.19 29.70	< 0.001 < 0.001 < 0.001
	Exp.	1.75 ± 0.98	3.48 ± 0.88	4.10 ± 0.74			
*Management of chemotherapy extravasation*	Cont.	20.20 ± 8.65	22.35 ± 7.79	22.65 ± 7.56	Group Time Time x Group	37.31 46.70 25.97	< 0.001 < 0.001 < 0.001
	Exp.	18.48 ± 9.66	31.78 ± 6.70	35.90 ± 3.54			
*Implementation of management of chemotherapy extravasation*	Cont.	2.05 ± 1.15	2.07 ± 0.94	2.25 ± 0.87	Group Time Time x Group	63.87 49.83 39.93	< 0.001 < 0.001 < 0.001
	Exp.	1.78 ± 1.03	3.90 ± 0.87	4.10 ± 0.78			
**Self-Care Self-Efficacy**	Cont.	2.68 ± 1.16	2.73 ± 0.86	2.66 ± 0.80	Group Time Time x Group	41.40 45.00 46.27	< 0.001 < 0.001 < 0.001
	Exp.	2.25 ± 1.15	4.06 ± 0.76	4.5 ± 0.59			

Table 6 indicates that, when analyzing the total sample of participants (n = 80), knowledge of chemotherapy extravasation (r = 0.805, p < 0.001) and self-care self-efficacy (r = 0.851, p < 0.001) increased with increasing self-care ability. Furthermore, knowledge of chemotherapy extravasation (r = 0.721, p < 0.001) increased with increasing self-care self-efficacy. These correlations were computed across all participants, regardless of group assignment.

**Table 6:** Correlation of knowledge, self-care, and self-efficacy (N = 80).

Variable	Knowledge of Chemotherapy Extravasation	Self-care Ability	Self-Care Self-Efficacy
Knowledge of Chemotherapy Extravasation	1		
Self-care Ability	0.805**	1	
Self-Care Self-Efficacy	0.721**	0.851**	1

**p < 0.001.

#### Participants’ perceived effectiveness of digital technology applications with educational program.

The study examined the perceived effectiveness of digital technology applications among 38 participants over three-time points (Table 7). In the total scale of perceived effectiveness of digital technology applications, there was a significant increase from 40.55 ± 6.83 at baseline to 45.72 ± 3.82 at 42 days follow-up (F = 9.93, *p* < 0.001). In the subscale of perceived usefulness, participants’ perceived usefulness of the digital technology applications increased from 11.88 ± 2.60 at baseline to 13.70 ± 1.24 at 42 days follow-up (F = 8.69, *p* < 0.001). For perceived ease of use, the perceived ease of use improved significantly from 12.33 ± 2.14 at baseline to 13.70 ± 1.27 at 42 days follow-up (F = 6.93, *p* < 0.05). In attitude toward using, there was a notable positive change in participants’ attitude towards using the technology, from 8.28 ± 1.40 at baseline to 9.08 ± 0.80 at 42 days follow-up (F = 5.28, *p* < 0.05). Trust in digital technology applications showed a significant increase from 8.08 ± 1.69 at baseline to 9.25 ± 0.90 at 42 days follow-up (F = 9.83, *p* < 0.001).

**Table 7:** Perceived Effectiveness of Digital Technology Applications (N = 38).

Variable	Pretest	21 days	42 days	Source	F	*p*
	Mean ± SD					
**Perceived Effectiveness of Digital Technology Applications**	40.55 ± 6.83	42.48 ± 6.81	45.72 ± 3.82	Time	9.93	< 0.001
Perceived Usefulness	11.88 ± 2.60	12.73 ± 2.22	13.70 ± 1.24	Time	8.69	< 0.001
Perceived Ease of Use	12.33 ± 2.14	12.88 ± 2.24	13.70 ± 1.27	Time	6.93	0.002
Attitude Toward Using	8.28 ± 1.40	8.45 ± 1.50	9.08 ± 0.80	Time	5.28	0.007
Trust	8.08 ± 1.69	8.43 ± 1.69	9.25 ± 0.90	Time	9.83	< 0.001

### Qualitative results

Our findings focus on how participants and nurses experience digital extravasation education intervention. The qualitative analysis identified three overarching themes that encapsulate the impact of the educational intervention on patients undergoing chemotherapy: *moving forward to self-care readiness, promoting emotional resilience, and enhancing positive care environment.*

#### Moving forward to self-care readiness.

The digital education intervention was introduced to improve participants’ understanding of chemotherapy extravasation and enhance their self-care skills. This intervention fostered that trust by providing clear, accessible information and empowering patients to take an active role in their treatment. As a result, participants exhibited a significant increase in their knowledge and awareness of chemotherapy extravasation after engaging with the program. With improved understanding, patients reinforce their readiness for self-care, enabling them to take greater control of their well-being and actively participate in their treatment journey.

The participants described:

*“Yesterday, a patient was lying on the bed next to me receiving chemotherapy. When the infusion site started swelling, he didn’t notice because he had fallen asleep. Since I had already been instructed on this, I noticed it in time and acted quickly—I shut off the IV system and went to inform the nurses. Luckily, only a small amount had leaked out, and I caught it in time. The nurses handled it, applied some compresses, and that was it—he recovered fine without any serious issues.” (B5)*

The nurse shared:

*“When patients become more knowledgeable, they even help educate other patients. Some of them, after learning about their condition and treatment, confidently share their knowledge with others. They take pride in what they know and willingly guide fellow patients. Overall, they are much more informed. Many patients love to share what they’ve learned. In rooms with some patients, they often engage in conversations, discussing and exchanging knowledge. Especially when there are new patients with little prior understanding, the more experienced ones are eager to guide and support them.” (A3)*

#### Promoting emotional resilience.

Through digital interventions, participants experienced a noteworthy positive psychological transformation. Many individuals reported feeling more reassured, with reduced anxiety and fear about chemotherapy. Patients exhibited greater confidence in their treatment journeys, contributing to improved emotional resilience. This transformation fostered a sense of empowerment, with patients appearing more self-assured throughout their treatment. Therefore, it enabled them to make informed decisions and actively participate in their care.

The participants expressed,

*“After receiving counseling, my mental state has stabilized a bit. I understand more now, and I try to maintain a positive mindset so I don’t worry too much. And nurses provide a lot of encouragement. Whenever I have any concerns, I just ask, and they respond immediately. That helps me feel much less anxious” (C3).*

*“When I received this education program from the nurses, I felt that my knowledge was enhanced, helping me improve my treatment process and access information to prevent complications and protect my health…. Not only that, but during conversations with family members, other patients, and their relatives, I also share this knowledge.” (B5)*

The nurse shared,

*“Their mental well-being has improved—they are happier, more optimistic, and more confident. When they come to the hospital, they no longer feel afraid of the hospital environment or chemotherapy. They feel more confident.” (A3)*

#### Enhancing a positive care environment.

These interventions fostered meaningful interactions, strengthening not only nurse-patient relationships but also connections among patients and caregivers. Digital platforms enabled real-time communication, allowing patients to share experiences, offer encouragement, and seek guidance from others undergoing similar treatments. Additionally, participants could access educational resources anytime and anywhere, ensuring they remained well-informed about their treatment. Digital interventions facilitated continuous care by enabling healthcare providers to remotely monitor patients, offer timely guidance, and address concerns as they arose. This remote monitoring did not involve IV chemotherapy administration at home; rather, it focused on communication and early detection of issues through patients’ digital updates. Such timely exchanges helped nurses respond more quickly to side effects or adherence difficulties, thereby enhancing patient safety and confidence. This ongoing support helped patients feel more connected, empowered, and supported throughout their chemotherapy journey.

Some participants recounted,

*“When there is a QR code, it is more convenient. When I have free time, I read it ….” (B1)*

*“When I received this education program from the nurses, I felt that my knowledge was enhanced, helping me improve my treatment process and access information to prevent complications and protect my health…. Not only that, but during conversations with family members, other patients, and their relatives, I also share this knowledge.” (B5).*

*“The department is nice, and nurses are wonderful. They take great care of patients, providing thorough education for both patients and their families so that we fully understand everything... I have no further expectations.” (C3)*

The nurses explained,

*“Being part of the nursing intervention team allows me to be closer to patients and communicate with them more easily. It also helps me receive early reports from patients (even when they get home), enabling quicker intervention. This approach strengthens the connection between nurses and patients.” (A1)*

This sense of strengthened connection may have been partly influenced by the experimental context, where nurses were more actively engaged in structured communication with patients as part of the intervention. As another nurse reflected,

*“Honestly, I feel more at ease knowing that after each chemotherapy session, when patients are discharged, they can inform their condition to me quickly via Zalo. Before, I used to worry a lot, wondering if they were okay at home. If something went wrong and they couldn’t follow their treatment regimen, I’d feel guilty. Now, with them proactively reaching out, it reduces my stress and anxiety. Otherwise, I’d constantly feel uneasy, fearing they might not know how to handle a situation or whether they’d even think to contact me. Being part of this intervention group means I also provide consultations via Zalo, which actually makes me happier. When patients ask questions, it shows that they trust and need me.” (A3)*

## Discussion

The present study aimed to assess the impact of chemotherapy extravasation education, grounded in Orem’s self-care theory, on the knowledge, self-care capabilities, and self-efficacy of patients undergoing chemotherapy. The findings revealed no significant differences in the mean scores of the experimental and control groups before the educational intervention. Altogether, these results indicate the positive effects of Orem’s self‑care theory on increasing knowledge, self-care, and self-efficacy in various patients and under different medical conditions, which are also consistent with the results of various studies. Systematic reviews indicate that nurse-led interventions are the most effective strategy for enhancing cancer patient outcomes [10,11]. Various interventions based on Orem’s theory have shown positive influences during the transition from early stages to home care, significantly impacting cancer patients’ knowledge, symptom management, their self-care behaviors, and self-efficacy [9,15,22–24]. Nurses are crucial in providing education, emotional support, and personalized guidance to patients, which strengthens their capacity to manage their health conditions effectively. By integrating evidence-based self-care implementation into regular cancer care, healthcare providers can improve treatment adherence, reduce complications, and enhance the overall quality of life for patients undergoing chemotherapy.

The qualitative analysis findings underscore the essential role of digital education in enhancing patients’ self-care readiness, strengthening emotional resilience, and fostering a supportive care environment. Schick et al. mentioned that digital interventions positively impact the patient-healthcare provider relationship [25]. Additionally, a systematic review found that digital patient education contributes to increased knowledge, emotional well-being, and behavioral changes [26]. These findings are consistent with previous research emphasizing the value of patient-centered digital interventions in effective disease management, reinforcing their role in improving health outcomes and patient engagement [27,28].

A comprehensive strategy to support health education interventions should incorporate a multimodal approach that includes video, handbooks, face-to-face interactions, telehealth, and e-health solutions [10,11]. By integrating these modalities, the interventions can be more effective, patient-centered, and adaptable to diverse healthcare needs. Previous reviews have indicated that oncology patients intend to utilize digital technologies following their transition back home to enhance positive health outcomes and foster patient empowerment [29,30]. Despite the clear benefits of digital education in promoting self-care, a major challenge remains: the cost of developing and implementing digital programs, particularly in resource-limited healthcare systems. To address this issue, leveraging freely available digital tools and integrating artificial intelligence offers a cost-effective solution in our study. By incorporating free digital strategies and AI-driven solutions, healthcare systems can overcome financial barriers while expanding access to high-quality patient education. Digital platforms should complement traditional face-to-face education, ensuring continuous support and accessibility for patients as they transition from hospital to home care.

### Limitations

This study has several limitations that should be acknowledged. First, the qualitative component included in-depth interviews with only six participants, which limits the breadth of perspectives and transferability of findings. Although qualitative research does not seek statistical generalization, future studies could expand the number and diversity of participants to enhance data richness and representativeness. Second, the digital chemotherapy extravasation education program was less effective for certain patient groups, such as older adults, illiterate individuals, people with disabilities (e.g., blindness or hearing impairment), ethnic minorities with limited technological exposure, and low-income patients who lacked access to smartphones. These barriers highlight the importance of developing alternative or complementary educational approaches, including in-person counseling sessions, printed materials, and family-centered support strategies. Lastly, the study was conducted at a single hospital in Vietnam, which may limit generalizability to other healthcare contexts. Future research should involve multiple sites and larger, more heterogeneous samples to confirm these findings and optimize intervention scalability.

## Conclusion

In conclusion, the education regarding digital chemotherapy extravasation, grounded in Orem’s Self-care theory, highlights the transformative potential of digital education in equipping patients for self-care, reinforcing emotional resilience, and cultivating a supportive care environment. As digital technology continues to progress, its incorporation into healthcare education will remain crucial for enhancing patient autonomy and improving overall treatment outcomes experiences. Future research could explore the long-term impact of digital self-care education on patient outcomes and treatment adherence.

### Practice implications.

Educating patients on digital chemotherapy extravasation through Orem’s Self-Care Theory can enhance their skills in recognizing, preventing, and managing extravasation incidents. Additional research is suggested to refine digital education strategies and evaluate their long-term effects on patient self-care behaviors.

## Supporting information

S1 File TREND checklist for reporting nonrandomized evaluations. This checklist provides a completed TREND (Transparent Reporting of Evaluations with Nonrandomized Designs) statement used to ensure comprehensive reporting of the study’s design, methodology, and findings.
(PDF)

S1 Data Minimal anonymized dataset used for statistical analyses. This file contains the anonymized participant-level data used for all analyses presented in the manuscript, including demographic information and scores for knowledge of chemotherapy extravasation, self-care ability, self-care self-efficacy, and perceived effectiveness of digital technology applications.
(SAV)

## References

[1] Bray F, Laversanne M, Sung H, Ferlay J, Siegel RL, Soerjomataram I, et al. Global cancer statistics 2022: GLOBOCAN estimates of incidence and mortality worldwide for 36 cancers in 185 countries. CA Cancer J Clin. 2024;74(3):229–63. doi: 10.3322/caac.21834 38572751

[2] Wengström Y, Margulies A, European Oncology Nursing Society Task Force. European Oncology Nursing Society extravasation guidelines. Eur J Oncol Nurs. 2008;12(4):357–61. doi: 10.1016/j.ejon.2008.07.003 18765210

[3] Anand U, Dey A, Chandel AKS, Sanyal R, Mishra A, Pandey DK, et al. Cancer chemotherapy and beyond: Current status, drug candidates, associated risks and progress in targeted therapeutics. Genes Dis. 2022;10(4):1367–401. doi: 10.1016/j.gendis.2022.02.007 37397557 PMC10310991

[4] Kim JT, Park JY, Lee HJ, Cheon YJ. Guidelines for the management of extravasation. J Educ Eval Health Prof. 2020;17:21. doi: 10.3352/jeehp.2020.17.21 32668826 PMC7431942

[5] Billingham MJ, Mittal R. Peripheral venous extravasation injury. BJA Educ. 2023;23(2):42–5. doi: 10.1016/j.bjae.2022.11.002 36686886 PMC9845539

[6] Onesti MG, Carella S, Fioramonti P, Scuderi N. Chemotherapy Extravasation Management: 21-Year Experience. Ann Plast Surg. 2017;79(5):450–7. doi: 10.1097/SAP.0000000000001248 28906302

[7] Melo JMA, Oliveira PP de, Souza RS, Fonseca DF da, Gontijo TF, Rodrigues AB. Prevention and conduct against the Extravasation of antineoplastic chemotherapy: a scoping review. Rev Bras Enferm. 2020;73(4):e20190008. doi: 10.1590/0034-7167-2019-0008 32578734

[8] Antúnez-Blancat A, Gago-Valiente F-J, García-Iglesias J-J, Merino-Navarro D. The Role of Nursing in the Management of Chemotherapy Extravasation: A Systematic Review Regarding Public Health. Healthcare (Basel). 2024;12(14):1456. doi: 10.3390/healthcare12141456 39057599 PMC11276807

[9] Rakhshani T, Najafi S, Javady F, Taghian Dasht Bozorg A, Mohammadkhah F, Khani Jeihooni A. The effect of Orem-based self-care education on improving self-care ability of patients undergoing chemotherapy: a randomized clinical trial. BMC Cancer. 2022;22(1):770. doi: 10.1186/s12885-022-09881-x 35840918 PMC9284903

[10] Bonetti L, Tolotti A, Anderson G, Nania T, Vignaduzzo C, Sari D, et al. Nursing interventions to promote patient engagement in cancer care: A systematic review. Int J Nurs Stud. 2022;133:104289. doi: 10.1016/j.ijnurstu.2022.104289 35751947

[11] Lanfear C, Harding S. The effectiveness of nurse-led care in supporting self-management in patients with cancer: A systematic review. J Clin Nurs. 2023;32(23–24):7996–8006. doi: 10.1111/jocn.16895 37837253

[12] Tanaka M. Orem’s nursing self-care deficit theory: A theoretical analysis focusing on its philosophical and sociological foundation. Nurs Forum. 2022;57(3):480–5. doi: 10.1111/nuf.12696 35037258

[13] Hartweg DL, Metcalfe SA. Orem’s Self-Care Deficit Nursing Theory: Relevance and Need for Refinement. Nurs Sci Q. 2022;35(1):70–6. doi: 10.1177/08943184211051369 34939484

[14] Li X, Zhang K, Xu D, Xu Y. The effect of Orem’s nursing theory on the pain levels, self-care abilities, psychological statuses, and quality of life of bone cancer patients. Am J Transl Res. 2023;15(2):1438–45. 36915789 PMC10006768

[15] Urtekin D, Eroglu SA. Effect of training based on Orem’s self-care deficit theory on breast cancer patients’ management of chemotherapy-related side effects and self-care behaviors: A randomized controlled trial. Eur J Oncol Nurs. 2024;73:102698. doi: 10.1016/j.ejon.2024.102698 39395233

[16] Kirsch EP, Kunte SA, Wu KA, Kaplan S, Hwang ES, Plichta JK, et al. Digital Health Platforms for Breast Cancer Care: A Scoping Review. J Clin Med. 2024;13(7):1937. doi: 10.3390/jcm13071937 38610702 PMC11012307

[17] Tudor Car L, Poon S, Kyaw BM, Cook DA, Ward V, Atun R, et al. Digital Education for Health Professionals: An Evidence Map, Conceptual Framework, and Research Agenda. J Med Internet Res. 2022;24(3):e31977. doi: 10.2196/31977 35297767 PMC8972116

[18] Kreidieh FY, Moukadem HA, El Saghir NS. Overview, prevention and management of chemotherapy extravasation. World J Clin Oncol. 2016;7(1):87–97. doi: 10.5306/wjco.v7.i1.87 26862492 PMC4734939

[19] Riegel B, Barbaranelli C, Sethares KA, Daus M, Moser DK, Miller JL, et al. Development and initial testing of the self‐care of chronic illness inventory. Journal of Advanced Nursing. 2018;74(10):2465–76. doi: 10.1111/jan.13775 29943401

[20] Zhou L, Bao J, Setiawan IMA, Saptono A, Parmanto B. The mHealth App Usability Questionnaire (MAUQ): Development and Validation Study. JMIR Mhealth Uhealth. 2019;7(4):e11500. doi: 10.2196/11500 30973342 PMC6482399

[21] Bengtsson M. How to plan and perform a qualitative study using content analysis. NursingPlus Open. 2016;2:8–14. doi: 10.1016/j.npls.2016.01.001

[22] Deng Q, Kang L, Zhu S, Luo W, Qing J, Zhong S, et al. Effects of nursing based on Orem’s self-care model on self-care efficacy, quality of life and adverse emotions in patients with advanced lung cancer. Am J Transl Res. 2021;13(4):2983–9. 34017465 PMC8129333

[23] Khoramabad NN. Implementation of Orem’s self-care model in early stages of breast cancer. Indian J Cancer. 2022;59(1):142–3. doi: 10.4103/ijc.IJC_1115_20 35645059

[24] Li S-Q, Luo C-L, Qiu H, Liu Y-X, Chen J-M. Effect of Orem’s self-care model on discharge readiness of patients undergoing enterostomy: A randomized controlled trial. Eur J Oncol Nurs. 2024;70:102549. doi: 10.1016/j.ejon.2024.102549 38692158

[25] Schick TS, Höllerl L, Biedermann T, Zink A, Ziehfreund S. Impact of Digital Media on the Patient Journey and Patient-Physician Relationship Among Dermatologists and Adult Patients With Skin Diseases: Qualitative Interview Study. J Med Internet Res. 2023;25:e44129. doi: 10.2196/44129 37738078 PMC10559188

[26] Schnitman G, Wang T, Kundu S, Turkdogan S, Gotlieb R, How J, et al. The role of digital patient education in maternal health: A systematic review. Patient Educ Couns. 2022;105(3):586–93. doi: 10.1016/j.pec.2021.06.019 34183217

[27] Marcu G, Ondersma SJ, Spiller AN, Broderick BM, Kadri R, Buis LR. The Perceived Benefits of Digital Interventions for Behavioral Health: Qualitative Interview Study. J Med Internet Res. 2022;24(3):e34300. doi: 10.2196/34300 35353045 PMC9008533

[28] Harrison Ginsberg K, Babbott K, Serlachius A. Exploring Participants’ Experiences of Digital Health Interventions With Qualitative Methods: Guidance for Researchers. J Med Internet Res. 2024;26:e62761. doi: 10.2196/62761 39607999 PMC11638693

[29] Jiang Y, Hwang M, Cho Y, Friese CR, Hawley ST, Manojlovich M, et al. The Acceptance and Use of Digital Technologies for Self-Reporting Medication Safety Events After Care Transitions to Home in Patients With Cancer: Survey Study. J Med Internet Res. 2024;26:e47685. doi: 10.2196/47685 38457204 PMC10960221

[30] Tuominen L, Leino-Kilpi H, Poraharju J, Cabutto D, Carrion C, Lehtiö L, et al. Interactive digital tools to support empowerment of people with cancer: a systematic literature review. Support Care Cancer. 2024;32(6):396. doi: 10.1007/s00520-024-08545-9 38816629 PMC11139693

